# Prospective environmental burdens and benefits of fast-swing direct air carbon capture and storage

**DOI:** 10.1038/s41598-024-66990-2

**Published:** 2024-07-17

**Authors:** Anne B. Ottenbros, Rosalie van Zelm, Jasper Simons, Mitchell K. van der Hulst, Kiane de Kleijne, Hans de Neve, Mark A. J. Huijbregts

**Affiliations:** 1 Department of Environmental Science, Radboud Institute for Biological and Environmental Sciences, Radboud University, P.O. Box 9010, 6500 GL Nijmegen, The Netherlands; 2 Carbyon BV, High Tech Campus 27, 5656 AE Eindhoven, The Netherlands; 3 Expertise Group Circularity and Sustainability Impact, TNO, P.O. Box 80015, 3508 TA Utrecht, The Netherlands; 4 Technology, Innovation and Society Group, Department of Industrial Engineering and Innovation Sciences, Eindhoven University of Technology, P.O. Box 513, 5600 MB Eindhoven, The Netherlands

**Keywords:** Prospective life cycle assessment, Direct air capture, Carbon capture and storage, Environmental impact, Climate change, Climate change, Environmental impact

## Abstract

Direct air capture (DAC) in combination with storage of CO_2_ can lower atmospheric CO_2_ concentrations. This study investigates the environmental impact of a new fast-swing solid sorbent DAC system, including CO_2_ transport and storage, over its life cycle, using prospective life cycle assessment. This DAC technology is currently on technology readiness level 5 and is expected to operate on an industrial scale by 2030. The technology was upscaled to the industrial scale and future changes in the background over the lifetime of the system were included, such as electricity grid mix decarbonization. Environmental trade-offs for the new DAC system were assessed by comparing environmental benefits from CO_2_ sequestration with environmental burdens from production, operation and decommissioning. We considered three electricity generation configurations: grid-connected, wind-connected, and a hybrid configuration. We found net environmental benefits for all configurations and background scenarios for ecosystem damage and climate change. Net human health benefits were observed when the electricity grid decarbonizes quickly and without the use of a battery. The environmental benefits increase with decreasing electricity footprint and are comparable with other DAC technologies. This illustrates that the new DAC system can help to meet the climate goals.

## Introduction

Carbon dioxide removal (CDR) methods lower atmospheric carbon dioxide (CO_2_) concentration by removing and durably storing CO_2_ from the atmosphere in products or geological, terrestrial, or ocean reservoirs^1^. CDR is required to achieve net zero greenhouse gas (GHG) targets, and is a feature of all modelled scenarios limiting global warming to 2 °C or lower by 2100^1^. These methods include afforestation and reforestation, enhanced weathering, bioenergy with carbon capture and storage (BECCS) and direct air carbon capture and storage (DACCS)^1–3^. Recently, direct air capture (DAC) has seen an increase in advancement and deployment^4–8^. Most DAC technologies are still under development, with technology readiness levels (TRLs) varying between 1 and 9^4^. Currently, 18 DAC plants worldwide are operational, capturing approximately 10 kton CO_2_ per year^9^.

Currently, two main types of DAC technologies are operational, based on liquid sorbents and solid sorbents, respectively^4,10^. Liquid sorbent systems need relatively high temperatures (~ 900 °C) to regenerate the solvent, whereas the heat demand for regeneration of solid sorbents is generally lower as temperatures around 80–120 °C are sufficient^4,5^. Despite their higher temperature demand, a recent life cycle assessment (LCA) on DACCS showed that liquid sorbents results in lower environmental impacts than solid sorbents in five out of eight investigated categories^11^. Previous LCA studies found that the environment impact, and the potential of DACCS to achieve negative emissions, is mainly dependent on the systems’ energy consumption and supply^11–16^.

A new fast-swing solid sorbent-based DAC system, under development by the company Carbyon, is designed to capture more CO_2_ per time unit compared to other DAC technologies due to its fast capture and regeneration cycle. This fast-swing DAC technology is still in an early stage of technology development (TRL 5) and early identification of environmental improvement options is essential in the further development of this technology towards sustainable industrial scale applications.

Here, we evaluated the environmental impact of this new fast-swing solid sorbent DAC system on industrial scale. We go beyond the current state of the art by identifying prospective net environmental consequences, including the effects of developments in the background system, such as penetration of renewable energy sources in the electricity grid, over the full lifetime of this new DAC system. Net environmental consequences, accounting for both environmental burdens as well as benefits from atmospheric CO_2_ removal, are quantified for the endpoint impact categories (1) damage to human health, (2) damage to ecosystem quality, and (3) damage to resource availability, next to the common midpoint impact category climate change. We provide the full life cycle inventory of the DAC system in Supplementary Information 2. Finally, the environmental performance of the new DAC technology was compared with other DAC technologies.

## Materials and methods

The general workflow of LCA according to the ISO14040^17^ was followed and complemented with the prospective framework developed by van der Hulst et al.^18^.

### System description

The fast-swing DAC system uses solid activated carbon consisting of phenolic resin coated with potassium carbonate (K_2_CO_3_). After adsorption of CO_2_ from ambient air on the sorbent material (Eq. (1)), the container with sorbent material is closed and a vacuum is created. In vacuum, the sorbent material is heated up to 80–120 °C using heat from an integrated electric heat pump. Subsequently, CO_2_ is released and the sorbent material regenerated. Adsorption and regeneration of the sorbent for this fast-swing process takes roughly 5–20 min. No external heat source is needed in this technology. Eventually, the CO_2_ stream leaving the DAC system can be geologically sequestered. Two CO_2_ transportation modes are considered: pipeline transport and ship transport. Pipelines are a cost-effective mode of CO_2_ transport for large volumes (1.5MtCO_2_/year) and are competitive with ship and barge transportation^19^.

1 $${\text{CO}}_{2}+{\text{H}}_{2}\text{O}+ {\text{K}}_{2}{\text{CO}}_{3}\rightleftharpoons 2 {\text{KHCO}}_{3}$$

Carbyon aims to develop the fast-swing DAC system from TRL 5, i.e. component validation of the system in ambient air, to TRL 6, i.e. final engineering with a pilot plant, by the end of 2024^20,21^. Full industrial scale (TRL 9) is projected to be reached in 2030.

### Goal and scope

#### System boundaries

**Figure 1:**
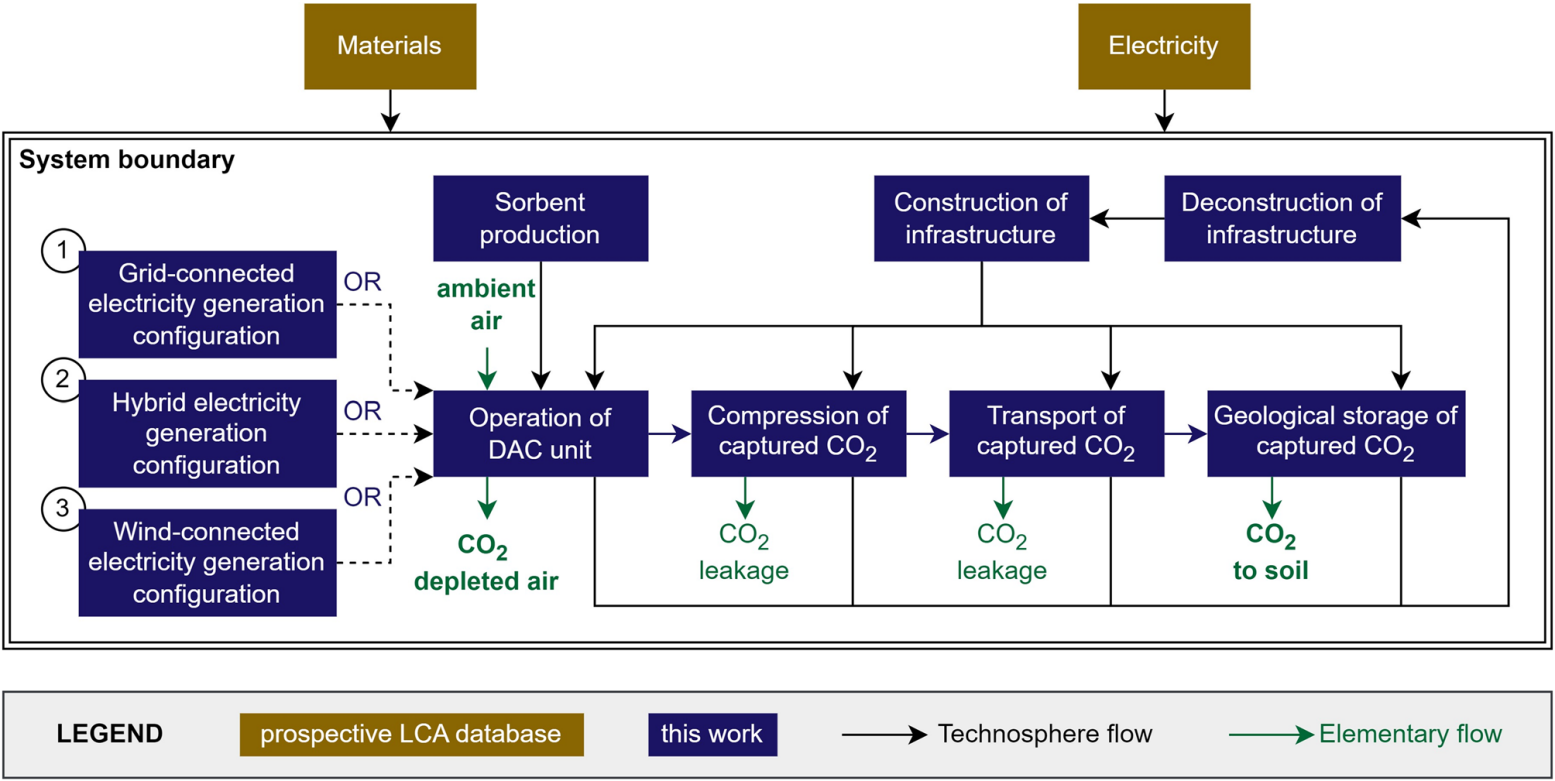
System boundaries of the direct air carbon capture and storage unit, including construction, operation and deconstruction. Electricity is supplied by wind and/or grid electricity.

Our study aims to quantify the environmental consequences of this DACCS system on industrial scale, considering annual changes to the background system throughout the lifetime of the system. The functional unit is “1 ton of CO_2_ captured from ambient air and geologically stored”. The study was performed with a cradle-to-grave perspective, so including end-of-life treatment of the DAC system and storage of the captured CO_2_ in a geological formation. Utilization options of the captured CO_2_ were not included in this study. The system boundary of this study includes the material in- and outputs, energy requirements and transport needed to build, operate, and dismantle a DAC unit (Fig. 1). Specifically, the replacement of the sorbent due to an expected lifetime of 3 years was included. Other types of maintenance were excluded, based on the expected negligible contribution^14^. The lifetime of a DAC unit was assumed to be 25 years, making the temporal scope 2030–2055. Processes were modelled to represent DAC placement in Western European coastal areas with CO_2_ storage in empty gas fields in the North Sea.

#### Electricity generation configurations

We consider three electricity generation configurations to meet the operational electricity need of the DAC system. The g*rid-connected configuration* assumes that the DAC system is connected to the grid and operates continuously. In the *hybrid configuration*, the DAC system is connected to a dedicated onshore wind park with a peak production capacity equal to that of the DAC system. The share of the year the DAC system operates on wind electricity is determined by its capacity factor, which is 0.23 in Western Europe^22^. Wind electricity is supplemented by grid electricity to ensure continuous operation. In the *wind-connected configuration*, the DAC system is off-grid and connected to a dedicated onshore wind park in combination with a lithium-ion (Nickel–Manganese–Cobalt) battery, based on the configuration in Terlouw et al.^23^. In this configuration, the wind park’s peak production capacity is double that of the DAC system, allowing the battery to charge during windy periods and power the DAC system when there is little or no wind. When the DAC system is already running and the battery fully charged, surplus wind electricity is curtailed. Due to the intermittency of wind and limited storage capacity of batteries^14,23^, an operation factor of 0.5 is assumed.

### Prospective assessment

The prospective assessment was carried out based on the prospective framework developed by Van der Hulst et al.^18^. Size scaling, process changes (e.g., efficiency increases in material and electricity use), and process synergies (e.g., heat recovery) occurring in upscaling from TRL 5 to TRL 9 were based on expert judgement from Carbyon and are described in the inventory and Supplementary Methods. The framework also prescribes the inclusion of external developments and industrial learning after the system reaches TRL 9. Industrial learning accounts for improvements in industrial production over time, which is not considered in this study, since impacts were derived for a first-of-a-kind industrially produced DAC unit. External developments during the system’s lifetime from 2030 to 2055 were included for the sectors of electricity generation, transportation, and production of steel, cement, and fuels modelled by integrating three future IAM scenarios with the background LCA database^24^.

In line with the state of the art in prospective LCA, narratives for the external developments were based on the shared socio-economic pathway (SSP)^25^ and representative concentration pathway (RCP)^26,27^ frameworks. This work considers SSP2, referred to as the “middle of the road” scenario, which assumes population, wellbeing, and technological developments following historic trends^26^. For SSP2, the baseline, RCP2.6, and RCP1.9 scenarios were assessed, corresponding with a global mean surface temperature rise of 3–4 °C, 1.6–1.8 °C, and 1.2–1.4 °C by 2100, respectively. These narratives were used by an integrated assessment model (IAM) to project future market developments. Outcomes from the IAM IMAGE^28^ were applied to the ecoinvent database version 3.9.1, system model “allocation cut-off by classification”^29,30^ using python package premise, version 2.0.2.^24^. Premise adjusts technology mixes for sectors modelled by IMAGE and stores a copy of the LCA database for each year and scenario considered. These copies were subsequently combined in a superstructure database and scenario difference file to streamline further scenario analyses^31^.

### Life cycle inventory

Detailed inventory data with projections on material composition and energy usage of the DAC system are provided in Supplementary Information 2. Since IMAGE has a lower spatial granularity than ecoinvent, dividing the world into 26 regions^28^, processes were defined for the region ‘Western Europe’ in line with our geographical scope.

#### Construction of DAC unit

Material and energy requirements on industrial scale are presented in Table 1. Each component was assumed to have a lifetime of 25 years, in line with other DAC studies^32^. Transport of each (sub-) component to the assembly location over a distance of 100 km and transportation of 100 km of the whole DAC installation from the assembly location to the site of installation was included as a default^33^. The energy mixture for the assembly processes was set to the assembly year (i.e., 2030).

**Table 1:** Life cycle inventory data for the construction and deconstruction per ton CO_2_ captured.

Component	Process inputs and outputs	Amount per one DAC unit over 25 years	Unit
Air pre-treatment	Plastics (50% PET & 50%PP)	5	Kg
	Steel	167	Kg
Air intake + transport	Steel	200	Kg
Cartridge	Steel	818	Kg
	Aluminium	20	Kg
	Cables	20	Kg
Vacuum vessel	Steel	578	Kg
Vacuum system	Plastics (50% PET & 50% PP)	5	Kg
	Steel	210	Kg
H_2_O treatment	Aluminium	130	Kg
	Steel	60	Kg
CO_2_ capture	Sensors	5	Kg
	Steel	100	Kg
Air outlet	Steel	110	Kg
Base frame + housing	Steel	1770	Kg
Electronics hardware	Solid state relay	200	Kg
	Printed wiring board	30	Kg
	Electronics	10	Kg
	Steel	190	Kg
Infrastructure around DAC	Asphalt	1266	Kg
	Broken rubble	2531	Kg
	Concrete	382	Kg
	Cables	2	Kg
	Steel	9	Kg
Assembly DAC unit	Electricity	24	kWh
	Transport	500	tkm

The sorbent material is not listed here, as it is replaced every 3 years during operation. The components are estimated to have a lifetime of 25 years.

The inventory data for the construction of the lithium-ion (NMC) battery pack and balance of system (BOS) for the wind-connected configuration was based on Schmidt et al.^34^. The BOS includes all hardware (e.g., inverter), software (e.g., battery and energy management systems) and services (e.g. construction)^34^. The construction year was set to 2030.

#### Operation DAC unit

The capture capacity of the DAC system is 100 ton CO_2_/year. The sorbent material of 250 kg was expected to need replacement to sustain this capture capacity every 3 years, resulting in a consumption of 0.8 kg sorbent/ton CO_2_ captured. Each sorbent replacement was modelled with the corresponding replacement year from the prospective LCA background database. During operation, water from ambient air is captured on the sorbent material. Roughly, 250 L water/ton CO_2_ is captured and released back to the environment. Additionally, each DAC machine is estimated to use 32 m^2^ area of land and can be placed in an industrial area.

The current (TRL 5) electricity consumption is 2500 kWh/ton CO_2_ captured, which is projected to be reduced to 1500 kWh by 2030 on industrial scale (TRL 9). Reduction from 2500 to 1500 kWh is expected to be realized by implementing an integrated heat pump to provide the necessary heat for CO_2_ to desorb and increase efficiency by making use of the heat present in the air that is processed inside the chamber.

The environmental impact of the generated electricity in the hybrid configuration (EI_elec,hybrid_) per kWh was calculated by Eq. (2). CF is the capacity factor of 0.23^22^. EI_wind_ is the environmental impact of wind electricity per kWh, corresponding to a wind farm with > 3 MW capacity in ecoinvent 3.9.1. EI_grid_ is the environmental impact of the grid electricity per kWh taken from the prospective database with the corresponding background scenario.

2 $${EI}_{elec, hybrid}=CF\cdot {EI}_{wind}+(1-CF)\cdot {EI}_{grid}$$

For the wind-connected configuration, the capacity of the DAC system (cap_DAC system_) in MW was calculated to be 17 MW following Eq. (3). M_CO2_ is the yearly CO_2_ capture rate of the DAC plant in ton CO_2_/year, and assumed to be 100 kton CO_2_/year^14,15^. P_DAC_ is the electricity requirement of the DAC system in kWh/ton CO_2_ captured and equal to 1500 kWh/ton CO_2_. The utilization factor (f_utilization_) of the system is equal to 1 for continuous operation (i.e., scenario 1 and 2), and 0.5 in the wind-connected configuration^23^.

3 $${cap}_{DAC system}=\frac{{M}_{{CO}_{2}}\cdot {P}_{DAC}}{{f}_{utilization}\cdot 8760 h}$$

The environmental impact of the battery per ton CO_2_ captured (EI_battery,tCO2_) was calculated according to Eq. (4). Cap_battery_ is the capacity of the battery, expressed in hours of electricity for full operation of the DAC system, and was assumed to be between 3 and 12 h^14^. In the base case, 6 h was assumed. LT_DAC_ is the lifetime of the DAC system of 25 years and EI_battery,kWh_ is the environmental impact of the battery expressed per kWh. LT_battery_ is the lifetime of the battery, assumed to be 20 years^34^.

4 $${EI}_{battery,{ tCO}_{2}}=\frac{{cap}_{DAC system}\cdot {cap}_{battery}}{{M}_{{CO}_{2}}\cdot {LT}_{DAC}}\cdot {EI}_{battery, kWh}\cdot \frac{{LT}_{DAC}}{{LT}_{battery}}$$

The environmental impact of the electricity generated in the wind-connected configuration (EI_elec,wind-conf_) per kWh was calculated according to Eq. (5). f_curtailment_ is the curtailment factor and represents the share of electricity that is curtailed, which needs to be accounted for in the total production of electricity, and depends on the hourly availability of wind electricity, and battery capacity and losses. f_curtailment_ varies between 0.2 and 0.7 for renewables-powered electrolysis processes^23^ and was assumed to be 0.5 in our scenario. The second argument of Eq. (5) accounts for compensating the electricity loss from the 90% battery round trip efficiency (e_battery_)^34^. This applies only to the share of electricity passing through the battery (f_battery_). The theoretical maximum of f_battery_ is 0.5, indicating that the gross electricity production increase is at most 5%. However, due to variability in wind availability, we assumed an f_battery_ of 0.25.

5 $$EI_{{elec,wind - conf}} = \frac{{P_{{DAC}} \cdot EI_{{wind}} }}{{1 - f_{{curtailment}} }} \cdot \left( {1 + f_{{battery}} \cdot \left( {\frac{1}{{e_{{battery}} }} - 1} \right)} \right)$$

#### Sequestration of captured CO_2_

The CO_2_ stream leaving Carbyon’s DAC system was assumed to be geologically stored underneath the North Sea (Porthos)^35^. The inventory for CO_2_ compression, pipeline transport and injection into wells was based on Koornneef et al.^33^ (Supplementary Information 2). Specifically, compression from 1 to 110 bar requires 111 kWh/ton CO_2_, and injection requires 7 kWh/ ton CO_2_. Ship transportation, consisting of compression to 6.5 bar and − 52 °C, loading and unloading, has a similar electricity demand of 118 kWh/ton CO_2_ (Supplementary Information 2)^36^. The transport distance to the injection well was assumed to be 100 km. CO_2_ leakage of 0.03% for compression and transportation was included^36,37^, but CO_2_ leakage after injection was assumed to be negligible^37^.

#### End of life treatment of DAC unit

Metals and electronics are recycled and the sorbent material is incinerated. Following the cut-off approach, the impact of breaking down the machine to separate components was included, but the burdens and benefits of recycling were not^29^. The processes for end-of-life treatment were modelled with an energy mixture of the year of disassembly (i.e., 2054). End-of-life of the battery system was not included, due to lack of data^34^.

#### Benefits of capturing CO_2_

For climate change, the benefit of capturing and storing atmospheric CO_2_ equals to 1 ton of CO_2-eq_ per functional unit. For human health and ecosystem quality this is respectively the reduction in disability adjusted life years (DALY) and species year lost by 1 ton of CO_2_ captured^38^. For the ReCiPe hierarchist perspective, this results in savings of 9.2810^−4^ DALY and 2.8010^−6^ species*year per ton of CO_2_.

### Life cycle impact assessment

Impacts were calculated using Activity Browser, a user interface for Brightway2. The life cycle impact assessment method ReCiPe 2016 v1.03 was used to calculate the environmental impact on endpoint level for damage to ecosystem quality, damage to human health, and damage to resource availability and on midpoint level for climate change^38,39^. The endpoint level was chosen to better understand potential burden-shifting between impact categories, like land use, fine particulate matter or water depletion^14–16^. The category climate change was included specifically, as capturing CO_2_ by DAC(CS) is relevant for this indicator and it enables comparison with other DAC studies^3,12,14–16^. Additionally, a contribution analysis indicating how much each midpoint contributes to each of the three endpoints was performed.

ReCiPe contains three cultural perspectives; egalitarian (E) considering short-term interest and technological optimism, hierarchist (H) representing a consensus perspective and individualist (I) representing a precautionary perspective with long time horizons^38^. Presented results assume the hierarchist perspective, with findings for other perspectives provided in the Supplementary Information 1.

Climate change impact categories in the default ReCiPe 2016 impact assessment method were adapted at both the midpoint and endpoint level to include characterization factors for the elementary flows of CO_2_ extracted from air and injected to soil (Supplementary Methods). These flows are a result of the introduction of CDR technologies in time. Furthermore, characterization factors were added for fugitive hydrogen emissions, in line with Sand et al.^40^, and were adjusted for biogenic methane emissions, in line with Muñoz and Schmidt^41^.

### Sensitivity analysis

To test the effect of assumptions and uncertainties in the prospective assessment, parameters related to the DAC system and electricity generation configurations were evaluated. The evaluated ranges for the DAC system were based on the reported inventory values for other DAC LCA studies and predictions from Carbyon.

1. Material usage of DAC construction was changed with 20% to test the uncertainty in size scaling of material requirements from TRL 5 to TRL 9.
2. The projected electricity consumption at TRL 9 (1500 kWh/ton CO_2_ captured) was varied between the electricity consumption at TRL 5 (2500 kWh) and 1000 kWh, in line with projected industrial scale electricity consumption of other solid-based DAC technologies^42,43^.
3. The lifetime of the DAC system was lowered from 25 years based on the projection of Carbyon to 20 years based on the projections for other DAC systems^15,16^.
4. The replacement frequency was shortened from 3 years to 1 year, resulting in a comparable sorbent consumption as Climeworks of 3.5 kg/ton CO_2_^15^. Additionally, a sorbent lifetime of 5 years is investigated to showcase the extremes^44^.
5. Varying the sorbent material performance, the total capture capacity was halved and multiplied by 1.5.
6. To test the influence of the sorbent type, the use of an alternative sorbent, amine on silica^11^, was investigated.

In addition, parameters within the electricity generation configurations were varied. For the hybrid configuration, the capacity factor for onshore wind in Western Europe was increased from 0.23 to 0.45^45^, resulting in a higher share of wind electricity compared to grid electricity. In the wind-connected configuration, the curtailment factor was varied between 0 and 0.7^23^, where a curtailment factor of zero represents a scenario where all surplus wind electricity is exported to the grid and used by another purchaser. In this configuration, the battery capacity was altered between 3 and 12 h^14^, and the share of electricity passing through the battery was changed between 0.1 and 0.5 (i.e., theoretical maximum).

## Results and discussion

### Prospective environmental impact of DACCS on industrial scale

**Figure 2:**
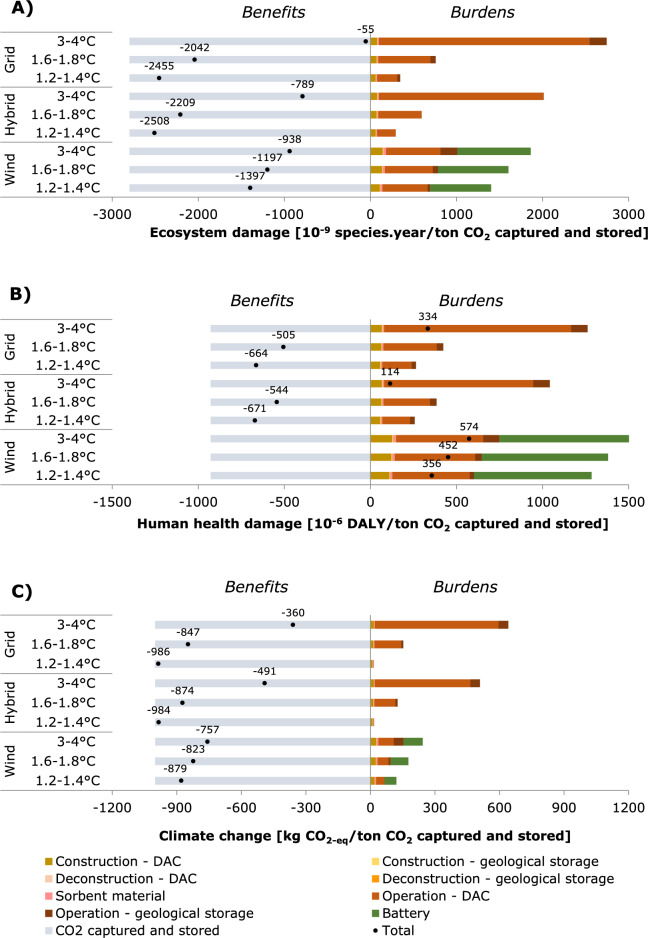
Prospective impact assessment results for climate change for the commercial scale DAC system build in 2030, for (**A**) damage to ecosystems in 10^−9^ species*year per ton CO_2_ captured and stored, (**B**) damage to human health 10^−6^ DALY per ton CO_2_ captured and stored and (**C**) climate change in kg CO_2-eq_ per ton CO_2_ stored and captured, calculated with ReCiPe 2016 Hierarchist perspective. Three scenarios are assessed: grid-connected, hybrid and wind-connected. For each scenario three prospective narratives are assessed; SSP2-RCP base (reaching 3–4 °C global warming by 2100), SSP2-RCP2.6 (reaching 1.6–1.8 °C global warming by 2100) and SSP2-RCP1.9 (1.2–1.4 °C global warming by 2100).

Figure 2 shows the burdens and benefits for ecosystem quality, human health, and climate change of capturing and storing 1 ton of CO_2_ using a fast-swing DAC system built in 2030 on industrial scale. The environmental benefits are larger than the burdens of building and operating the DACCS system for damage to ecosystems and climate change, regardless of electricity generation configuration and background scenario. Conversely, the human health benefits are only larger than the burdens for the grid-connected and hybrid electricity configurations, and only if external developments are in line with limiting warming to 2 °C or < 1.5 °C. The contribution analysis shows that climate change, human toxicity and fine particulate matter formation are the main impact categories contributing to damage to human health and ecosystems (Supplementary Fig. S1). The results these indicators and for damage to resource availability are shown in Supplementary Figs. S2 and S3, respectively.

Environmental impacts highly depend on the operational phase of the DAC system. The main contributor is the electricity consumption of the heat pump for CO_2_ desorption from the sorbent material. This finding is in line with other LCAs on sorbent-based DAC systems^4,13–16,46–48^. The environmental impacts associated with electricity consumption are determined by the grid mix composition specific to each background scenario, and the choice of electricity configuration. In the 3–4 °C background scenario, the grid-connected electricity configuration results in the highest impacts for the ecosystem damage and climate change indicators, followed by the hybrid configuration, due to a high share of fossil fuels in the grid mix. The lowest impacts are obtained for the wind-connected configuration with this background scenario. Only for the human health indicator does the wind-connected configuration have a higher impact, due to impacts from producing the battery. In the < 2 °C and < 1.5 °C background scenarios, the grid mix is increasingly based on renewables, with the latter seeing a faster implementation of renewables which even result in a negative emission intensity from 2040 onwards due to the inclusion of CDR methods (Supplementary Information 2). The environmental impacts associated with battery production play an even more pronounced role in these background scenarios: the wind-connected configurations with a battery consistently perform worst on all impact categories, while the hybrid configuration outperforms the grid-connected configuration.

The impacts from manufacturing of the DAC system, excluding sorbent material production, are relatively small compared to the total impacts for grid-connected DAC systems. However, for the wind-connected configuration and grid-connected configurations in the < 2 °C and < 1.5 °C background scenarios, the relative contribution from assembly becomes more prominent. This indicates that reduced material use becomes increasingly more important when DACCS is applied at the gigaton scale^1^. A hotspot analysis on the assembly phase (Supplementary Fig. S4) shows that a major contributor all indicators is the use of electronics in the hardware to control the system and operate it automatically, of which the printed wiring board contributes most. On industrial scale, the use of electronics should thus be carefully considered and be minimized where possible. The second major contributors are the steel used in the base frame and housing and the heat pump.

Changing to ship transport increased climate impacts with 25.4 kg CO_2-eq_/ton CO_2-eq_ captured and stored, ecosystem impacts by 0.8 10^−9^ species*year/ton CO_2_ captured and stored and human health impacts by 0.3 10^−6^ DALY/ton CO_2_ captured and stored. The relative change per configuration and background scenario is smaller for the latter two impact categories, as the main difference between the two transport modes is the higher level of CO_2_ leakage of 2.5%^49^.

The results for the egalitarian and individualist ReCiPe 2016 perspectives show a similar order in high to low impacts per electricity generation configurations for each background scenario (Supplementary Figs. S5–S6). However, ecosystem damage burdens are obtained if the system is grid- or hybrid connected in the 3–4 °C background scenarios. In the egalitarian perspective, wind electricity shows higher ecosystem impacts than grid electricity. This is predominantly caused by the use of chromium steel and its corresponding high characterization factor for ecosystem damage in the egalitarian perspective.

### Sensitivity analysis

**Figure 3:**
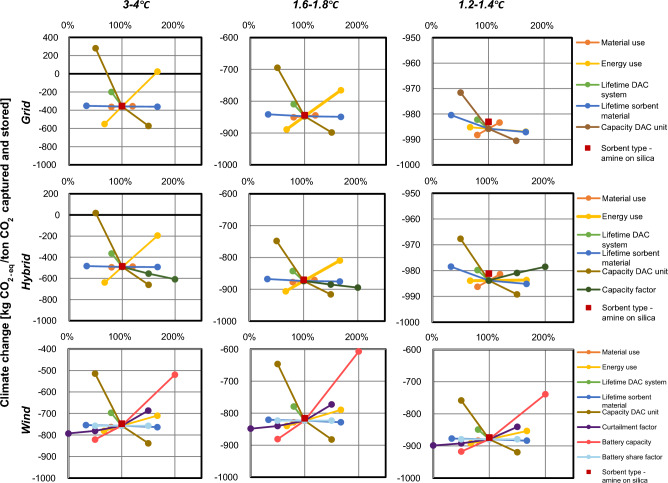
Sensitivity analysis for climate change in kg CO_2-eq_ per ton CO_2_ captured and stored on the most uncertain parameters in the prospective assessment for all three electricity generation configurations and all three background scenarios. The results are calculated with ReCiPe 2016 (H).

The results for the sensitivity analysis are shown for the climate change and all background scenarios (Fig. 3). Sensitivity analyses for the human health, ecosystem quality and resource availability indicators are provided in Supplementary Figs. S7–S9.

Varying the sorbent material’s capture capacity has the largest effect on absolute impacts, showing an inverse relationship to the net environmental consequence. For the 3–4 °C background scenario, net climate change burdens would occur for the grid- and hybrid connected system if the capture capacity on industrial scale is higher than projected. For human health and ecosystem damage net burdens occur for all electricity generation configuration in the 3–4 °C background scenario. Analogously, if the decrease in electricity consumption stagnates at the pilot scale (2500 kWh/ton CO_2_), climate change burdens would exceed climate change benefits in the grid-connected configuration in the 3–4 °C scenario. Although it is unknown whether the energy efficiency improvements will be achieved, the projected values for electricity consumption are in the same range as for Climeworks^14,15^.

Varying the material use, lifetime of the system and sorbent has a smaller effect on the net climate change impact. In the wind-connected configuration, with its utilization factor of 0.5, the effect is larger than for the other configurations because twice as many DAC systems are needed to capture the same quantity of CO_2_ per year. The system and sorbent lifetimes will mainly depend on the site of installation and corresponding weather conditions affecting the different DAC components. Changing the sorbent type to amine on silica increased the impact by less than 1%, due to the small amount used.

For a higher capacity factor of wind electricity, the net climate change impact in the hybrid configuration would be reduced by 2% in the 2 °C scenario, and by 24% in the 3–4 °C scenario. This reiterates the importance of weighing in the background grid mix in choosing between different electricity configurations.

The wind-connected configuration relies on a battery to supply electricity during part of the year, its capacity depending on the locally-specific wind generation profile^23^. While varying the share of electricity that passes through the battery did not have an effect, increasing the battery capacity to 12 h reduced the net climate change benefit by 26% in the 2 °C scenario. Still, the benefits remain considerable, regardless of the background scenario. However, for human health, the increased battery capacity increases the burden by 450% in the 2 °C scenario. Lastly, varying the curtailment factor in the 2 °C scenario between zero and 0.7 would result in a net climate change benefit decrease of 3% and increase of 6%, respectively.

### Comparison with other DAC systems

Table 2 shows the climate change impacts, excluding the benefits of storing, for capturing one ton of CO_2_ for other solid sorbent-based DAC studies. The carbon capture efficiency (CCE) is calculated with Eq. (6), where CO_2,captured_ is the quantity of CO_2_ captured from ambient air and GHG_capture process_ is the climate change emissions of construction, operation and deconstruction of the DAC system^13,15^.

**Table 2:** Climate change burdens and carbon capture efficiencies for solid-sorbent based DAC systems, reported by various LCA studies.

Source	Modelled TRL	Energy source	Climate change burdens [kg CO_2-eq_/ton CO_2_ captured]	Carbon capture efficiency (CCE) (%)
This study	Industrial scale	Grid-connected	14–640	36.0–98.6
	Industrial scale	Hybrid	16–509	49.1–98.4
	Industrial scale	Wind-connected	121–243	75.7–87.9
Deutz et al.^15^	Demonstration scale	Geothermal energy	69	93.1
	Demonstration scale	Grid electricity + heat from municipal waste incineration	146	85.4
	Industrial scale	Wind	120–50	88–95
Terlouw et al.^14^	Industrial scale	Fresnel & solar & battery	160–90	84–91
	Industrial scale	HTHP & solar & battery	110–210	79–89
	Industrial scale	HTHP & grid	50–910	9–95^a^
	Industrial scale	Waste heat & grid	60–520	48–94
	Industrial scale	Waste heat & solar & battery	80–150	85–92
Madhu et al.^16^	Demonstration scale	Low-carbon energy supply	140	86
Qiu et al.^11^	Industrial scale	Grid electricity (in 2020) & biomass or heat pump	60–640^b^	36–94
	Industrial scale	Grid electricity (in 2100) & biomass or heat pump	280–0^b^	72–112^c^
Casaban and Tsalaporta^48^	Industrial scale	Wind	110–130	87–89

^a^Dependent on the country specific grid mix.

^b^Also includes the impacts of DAC systems with solvent based sorbents.

^c^Value larger than 100% is due to a future grid electricity incorporating carbon capture technologies.

6 $$CCE= \frac{{\text{CO}}_{2, captured}-{GHG}_{capture process}}{{\text{CO}}_{2,captured}}$$

The performance of the fast-swing DAC system is comparable to other solid-sorbent based DAC systems on industrial scale. The relatively low CCE of 36% for a grid-connected DAC system in the 3–4 °C scenario is in line with CCE values found by Qiu et al.^11^ and Terlouw et al.^14^ for a grid-connected system. The large range obtained for the CCE in this study and literature shows the dependency of DAC performance on the environmental footprint of the supply of heat and electricity, and highlights the need for low-emission energy supply. Carbyon’s DAC system relies exclusively on electricity, and is thus not dependent on low-emission heat supply.

The upscaling of inventory data to industrial scale by Madhu et al.^16^ was done linearly and focused on the foreground data. We took this a step further by also adapting the background data to match the time scale of the foreground system. By not taking the change to a future, greener electricity mix into account for background processes, a mismatch occurs and CCEs are underestimated^18,50^. Qiu et al.^11^ and Terlouw et al.^14^ used a similar approach as our study in accounting for the mismatch in time for foreground and background to estimate the environmental impacts of a DAC system on industrial. They found a larger CCE for future scenarios, explained by not accounting for the changing grid mix during the DAC lifetime, as this study did.

### Future outlook

The fast-swing DAC system can be switched on and off within hours, which would make it possible to run on excess electricity at peak hours resulting from a high share of intermittent renewables in the grid mix. Daggash et al.^45^ found that this could achieve 0.23–0.67 ton CO_2_ avoided per MWh curtailed power for power-to-DAC systems. This could help to lower the competition for fossil-free electricity. Although excess electricity is considered a waste and thus has an environmental impact of zero based on economic allocation, exclusively using excess electricity would decrease operation time considerably compared to grid- or wind-connected systems and increase costs per ton of CO_2_ captured. Whether a decrease in operation time results in a substantial increase in lifetime is not yet proven.

As shown previously the location determines the net capture potential depending on temperature, humidity, air impurities (e.g. dust), availability of renewable electricity sources and potential storage sites^6,14^. Higher temperatures are expected to save electricity consumption by already increasing the air temperature, but simultaneously allows for quicker condensation on the sorbent material. As CO_2_ and water are competing for adsorption sites on the sorbent material, it is expected that a higher humidity results in less CO_2_ that can be captured. Dependencies in environmental variations still need to be evaluated for large scale DAC systems. For future plant construction, careful considerations of these environmental conditions with access to renewable energy and storage location should be made.

Evaluating future background changes during the DAC lifetime should be considered when performing prospective life cycle assessment. Depending on the speed of decarbonization in prospective socio-economic development scenarios, this can lead to large differences in the total impact of an emerging system. This is especially true for DAC systems, for which the total environmental impact is highly dependent on the impact of electricity demand over the full operation period.

### Supplementary Information

Supplementary Information 1.

Supplementary Information 2.

Supplementary Legends.

## Data Availability

All data generated or analysed during this study are included in this published article (and its Supplementary Information files).
