# Supplementary material for: Prospective environmental burdens and benefits of fast-swing direct air carbon capture and storage
Source: Sci Rep. 2024 Jul 17;14:16549. doi: 10.1038/s41598-024-66990-2 (PMC11255244; doi:10.1038/s41598-024-66990-2)
Supplement: Supplementary file 1 — Supplementary Information 1. [file 41598_2024_66990_MOESM1_ESM.pdf]

# Supplementary information 1

# Prospective environmental burdens and benefits of fast-swing direct air carbon capture and storage

Anne B. Ottenbros[1*], Rosalie van Zelm[1], Jasper Simons[2], Mitchell K. van der Hulst[1,3], Kiane de Kleijne[4] Hans de Neve[2], Mark A.J. Huijbregts[1,3]

[1] Department of Environmental Science, Radboud Institute for Biological and Environmental Sciences, Radboud University, P.O. Box 9010, 6500 GL, Nijmegen, The Netherlands.

[2] Carbyon BV, High Tech Campus 27, 5656 AE, Eindhoven, The Netherlands

[3] Expertise Group Circularity & Sustainability Impact, TNO, P.O. Box 80015, 3508 TA, Utrecht, The Netherlands

[4] Technology, Innovation and Society Group, Department of Industrial Engineering and Innovation Sciences, Eindhoven University of Technology, Eindhoven, The Netherlands

[*] Corresponding author: anne.ottenbros@ru.nl

# Supplementary methods

## Adjustments for ReCiPe 2016 in Activity Browser

In the prospective database created with premise [1], several scenarios were included: SSP2-RCP base (reaching 3-4 degrees global warming in 2100), SSP2-RCP2.6 (reaching 2 degrees global warming) and SSP2-RCP1.9 (1.5 degrees global warming). These scenarios rely on the inclusion on bioenergy with carbon capture and storage (BECCS), direct air capture (DAC) and other forms of biogenic or atmospheric $CO_2$ storage. These elementary flows are not yet quantified in the impact assessment methods and were added manually in Activity Browser. The characterization factors for these missing elementary flows are listed in Supplementary information 2, tab "adjusted CF ReCiPe2016". The characterization factors are added for each ReCiPe 2016 perspectives: Hierarchist, Egalitarian and Individualist [2].

For the uptake of $CO_2$ a negative characterization factor is added for climate change, damage to human health (climate change) and damage to ecosystems for both terrestrial ecosystems (climate change) and aquatic ecosystems (climate change). Non-fossil $CO_2$ release to the air is quantified with a positive characterization factor for these impact categories.

The characterization factors for biogenic methane should be reduced by 2.75 kg $CO_2$-eq compared to fossil methane, independent of the time horizon of the impact assessment method [3]. The factors for the elementary flows for biogenic methane have been adjusted accordingly.

Additionally, in some scenario's hydrogen-based supply chains are significant and the corresponding elementary flows were also added to the impact assessment method. Even though hydrogen itself is not a greenhouse gas, by its chemical reaction the abundance of other greenhouse gasses and aerosols are altered [4]. The characterization factors for a 20 (GWP20), 100 (GWP100) and 500 (GWP500) year horizon were added accordingly.

## Assumptions on size scaling, process synergies and synergies

*Size scaling*

Carbyon uses materials that are already available on the market and relatively cheap. The production of activated carbon has already been upscaled, as it is widely used for industrial filtration systems. The functionalization of the activated carbon with K2CO3 is done with a wet-chemical impregnation process that is already used for other purposes at large scale by the chemical industry. The technology is currently at TRL5 and evolves to TRL6 by end of 2024. The investment to reach TRL6 are estimated to be around 15M Euro. The next steps will be the mass manufacturing. Obviously substantial investments will be needed to build the production facilities by there is a strong business case and investors are willing to finance the production facilities given the huge demand for machines to capture CO2 from the atmosphere. Carbyon designed its DAC system as small units that it could be easily be stacked and scaled-up to a large capacity plant.

*Process changes and synergies*

Reduction from 6000 kWh per ton CO2 captured on lab scale to the current predicted 2500 kWh was realized by suppressing the condensation of water on the sorbent material, by shortening the adsorption time and slightly elevated temperatures (10 to 20 °C above room temperature). Reduction from 2500 kWh to 1500, is expected to be realized by implementing an integrated heat pump to provide the necessary heat for CO2 to desorb. The heat pump is expected to increase the efficiency, by making use of the heat present in the air that is processed inside the chamber.

# Supplementary Results

## Contribution analysis on endpoint level

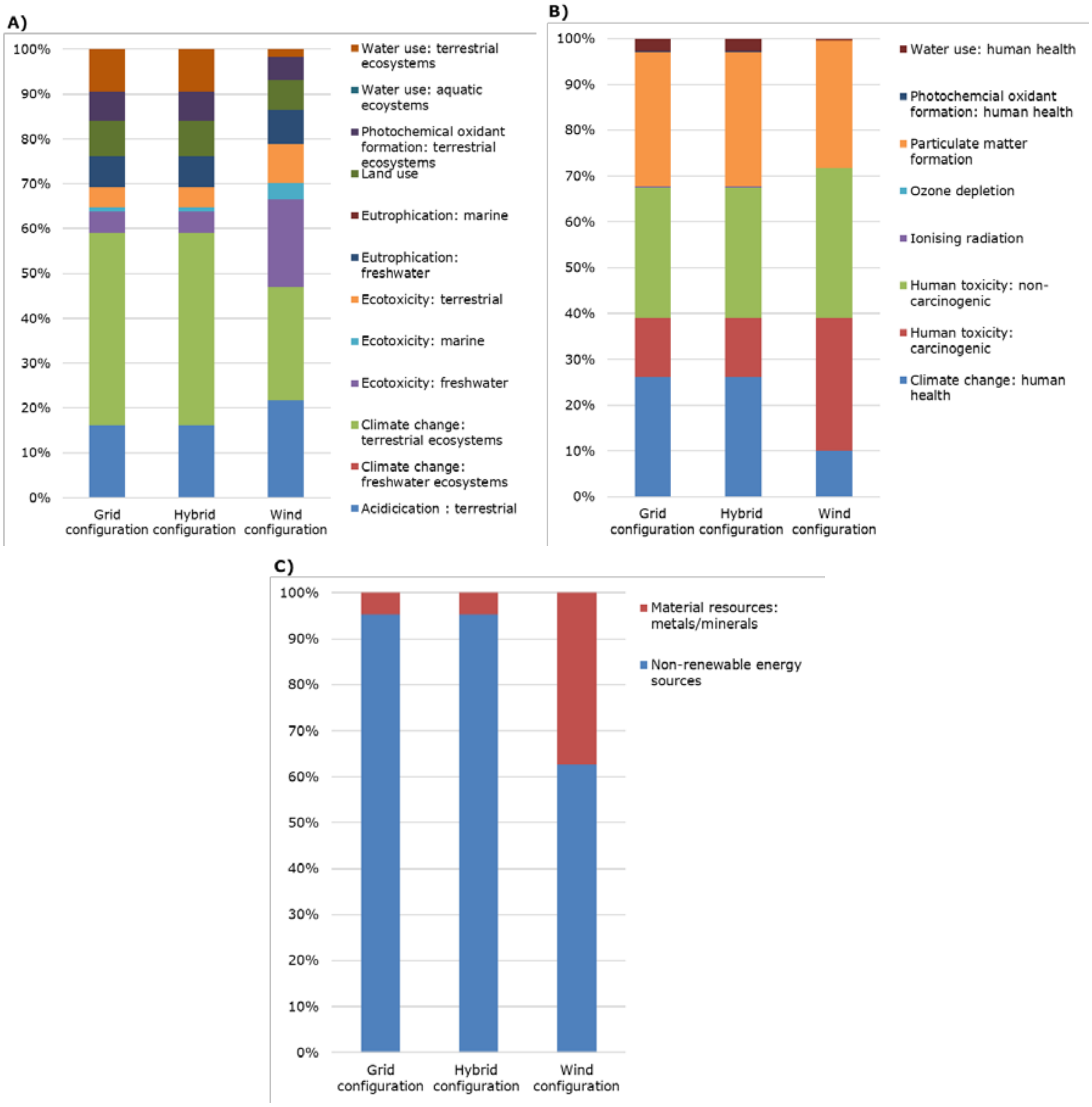


**Figure S1.** Midpoint to endpoint contribution analysis for A) damage to ecosystems, B) damage to human health, and C) damage to resource availability for the SSP2-RCP2.6 prospective scenario (<2°C global warming), calculated with ReCiPe 2016 (H).

## Results on resource damage

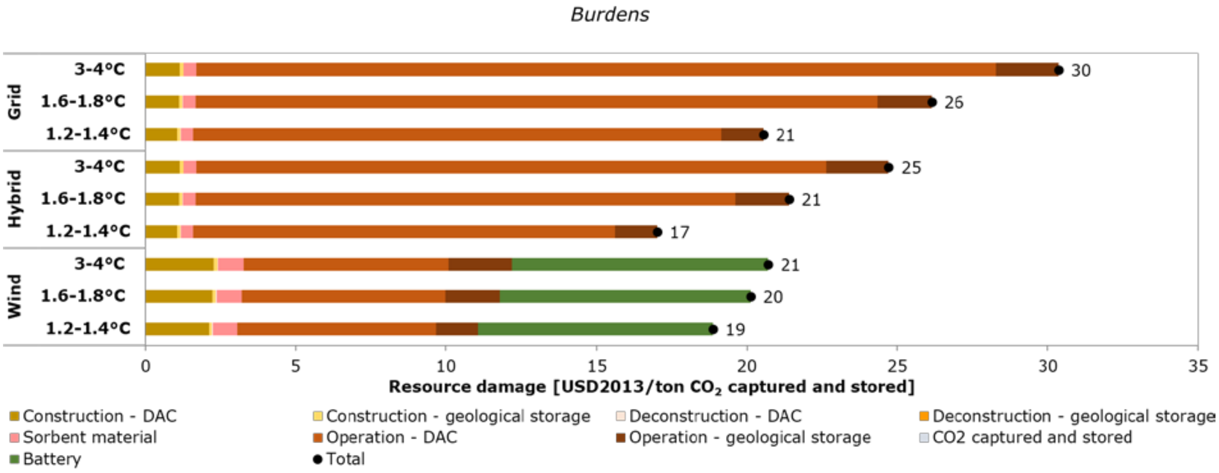


**Figure S2.** Prospective results for resource damage calculated with ReCiPe 2016 (H).

## Results on land use, fine particulate matter and human (non-)carcinogenic toxicity

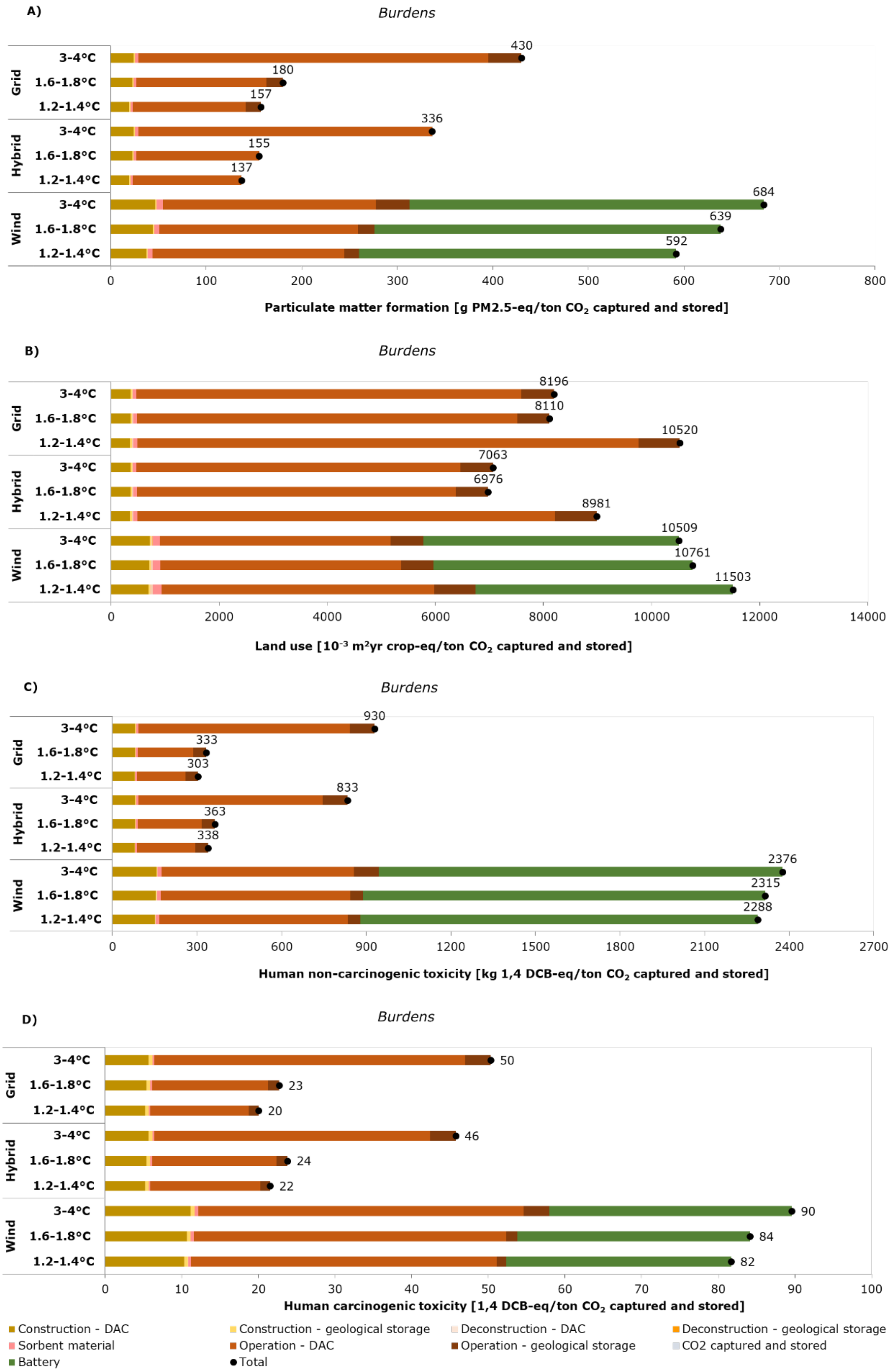


**Figure S3.** Results for A) fine particulate matter, B) land use, C) human non-carcinogenic toxicity and D) human carcinogenic toxicity, calculated with ReCiPe 2016 (H). These midpoints were selected based on the contribution to the three endpoints (Fig. S2). The land use impact increases for the <1.5°C background scenario, due to the inclusion of bioenergy carbon capture and storage (BECCS) in this scenario. BECCS requires most land of the carbon dioxide removal (CDR) methods.

## Contribution analysis on process level

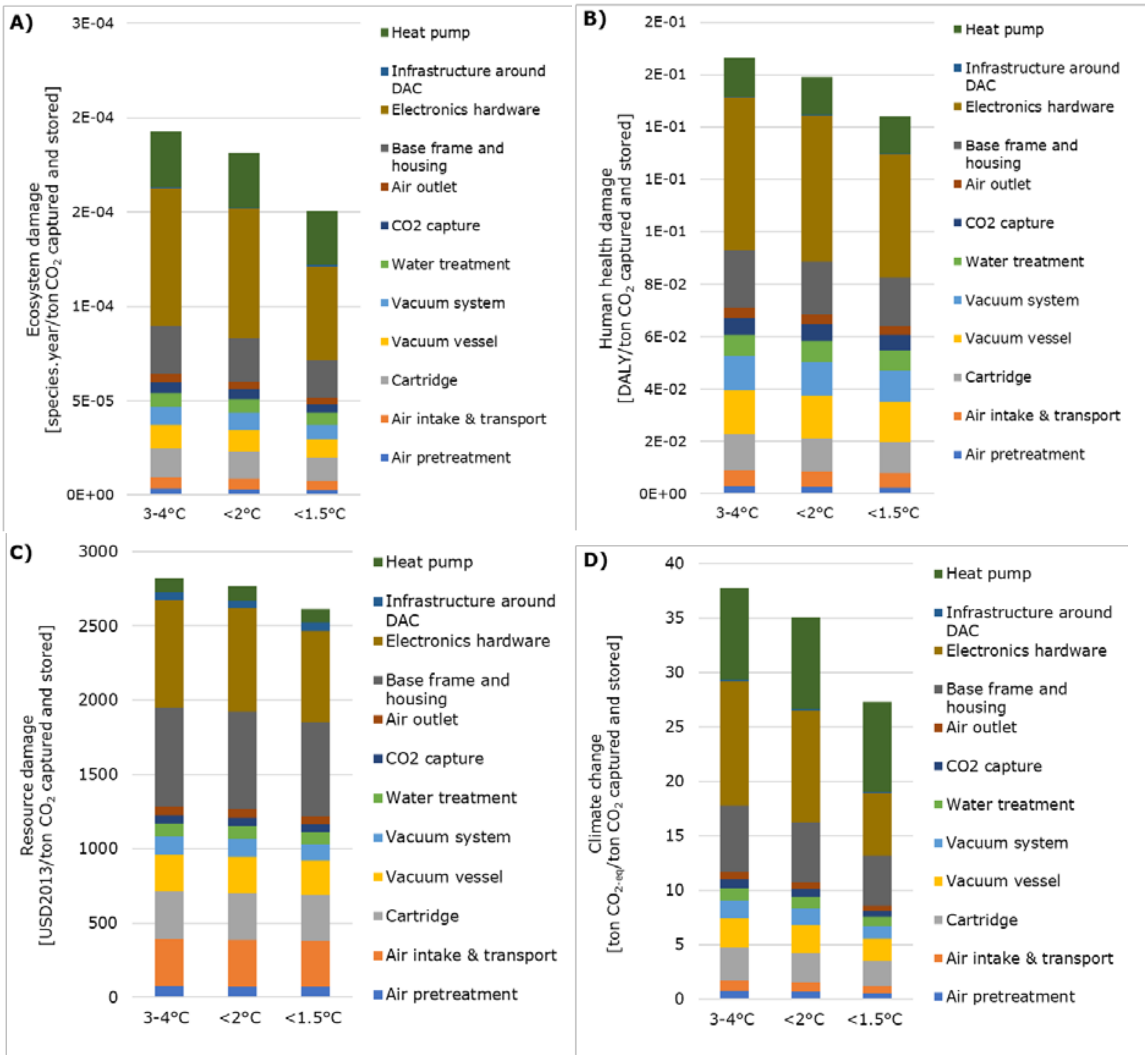


**Figure S4.** Contribution analysis on the material requirements of the DAC system (construction phase) for A) damage to ecosystems, B) damage to human health and C) damage to resource availability and D) climate change, calculated with ReCiPe 2016 (H).

## Prospective environmental impacts of DACCS on commercial scale

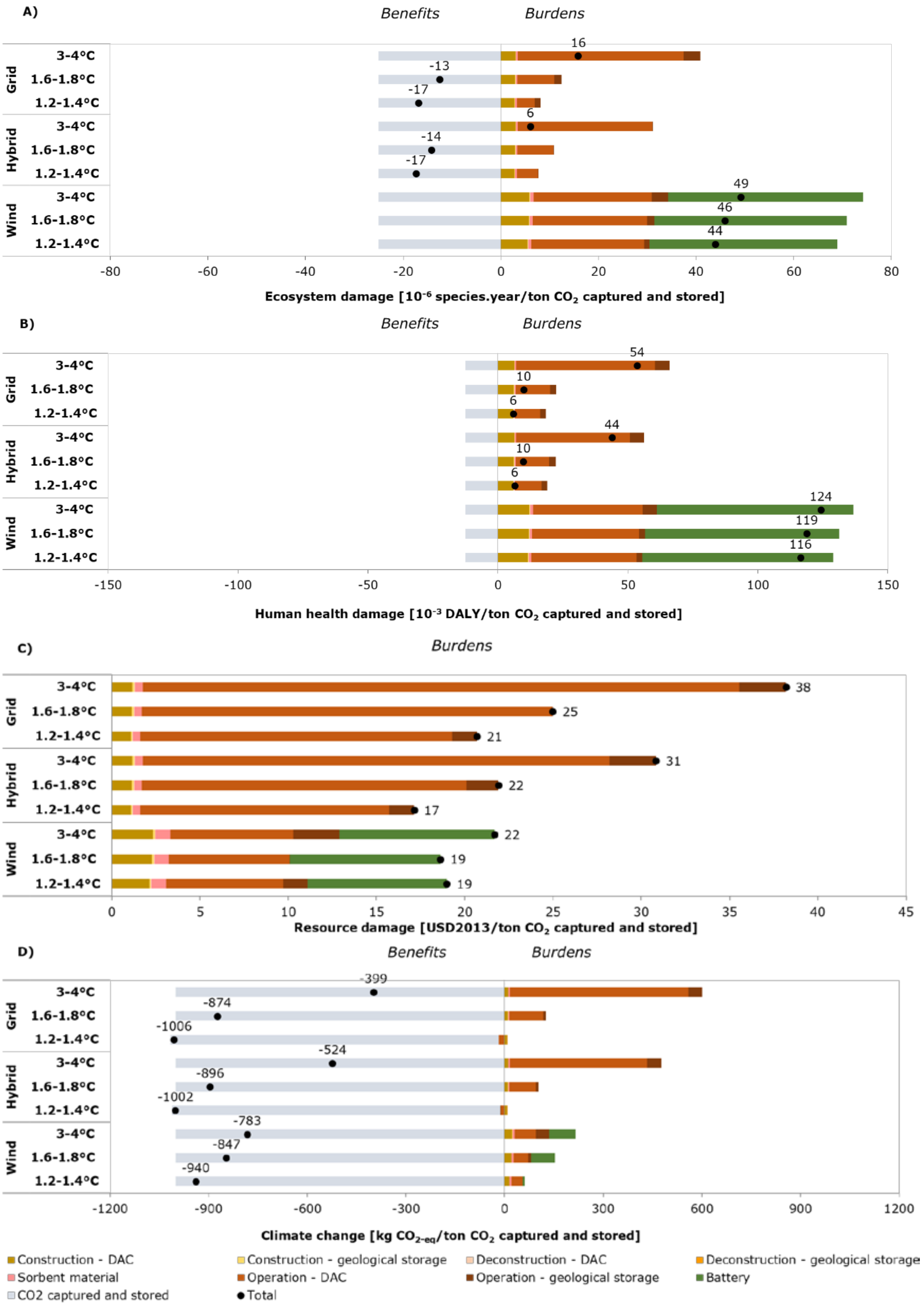


**Figure S5.** Prospective impact assessment results for the commercial scale DAC system build in 2030, for A) damage to ecosystems, B) damage to human health, C) damage to resource availability and D) climate change, calculated with ReCiPe 2016 Egalitarian perspective. Three electricity generation configurations are assessed: grid-connected, hybrid and wind-connected. Next to three configuration, three prospective scenarios are assessed; SSP2-RCP base (reaching 3-4°C global warming), SSP2-RCP2.6 (reaching 1.6-1.8°C global warming) and SSP2-RCP1.9 (1.2-1.4°C global warming).

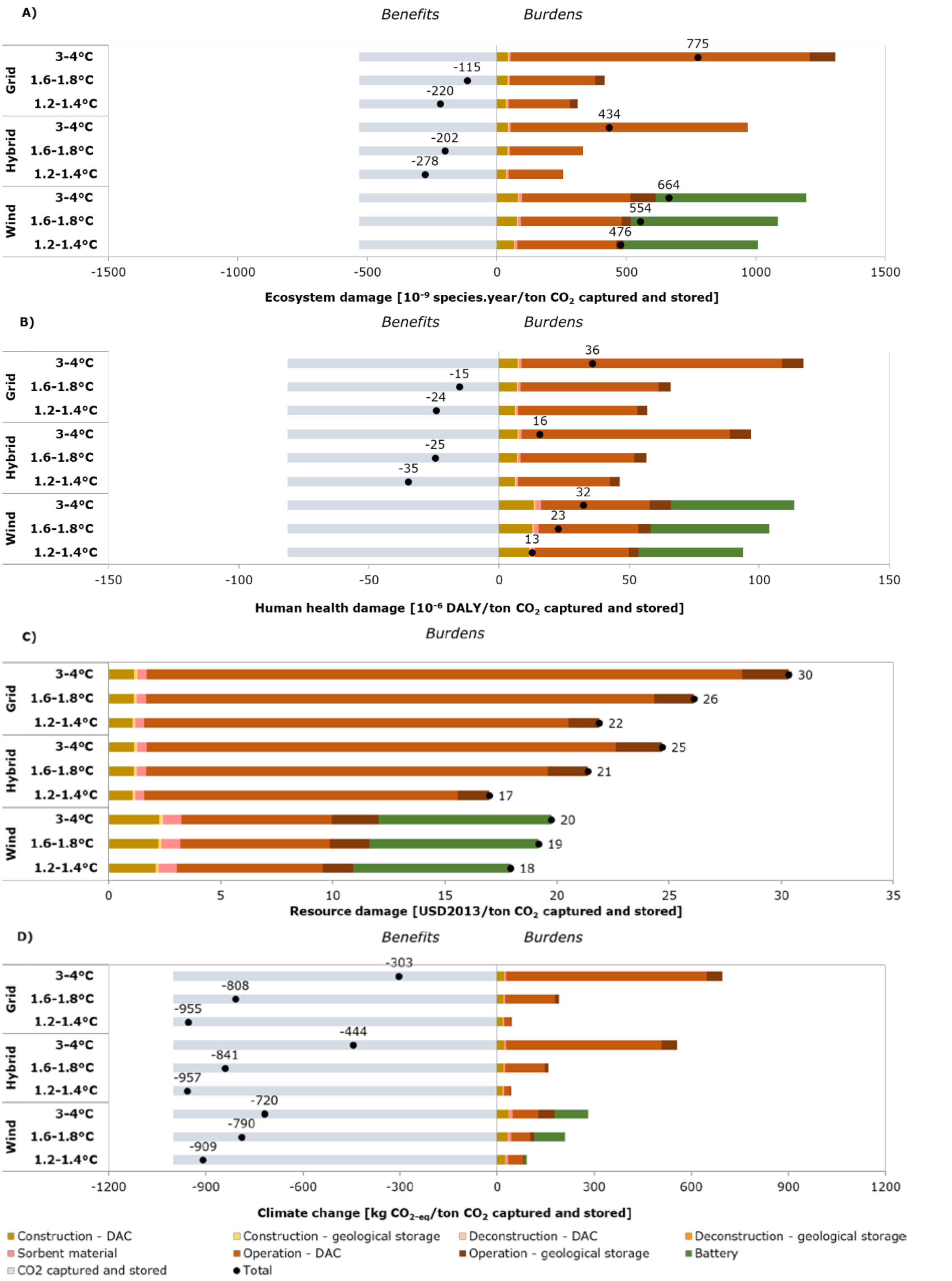


**Figure S6.** Prospective impact assessment results for the commercial scale DAC system build in 2030, for A) damage to ecosystems, B) damage to human health, C) damage to resource availability and D) climate change, calculated with ReCiPe 2016 Individualist perspective. Three electricity generation configurations are assessed: grid-connected, hybrid and wind-connected. Next to three configuration, three prospective scenarios are assessed; SSP2-RCP base (reaching 3-4°C global warming), SSP2-RCP2.6 (reaching 1.6-1.8°C global warming) and SSP2-RCP1.9 (1.2-1.4°C global warming).

## Sensitivity analysis for human health, ecosystem and resource damage

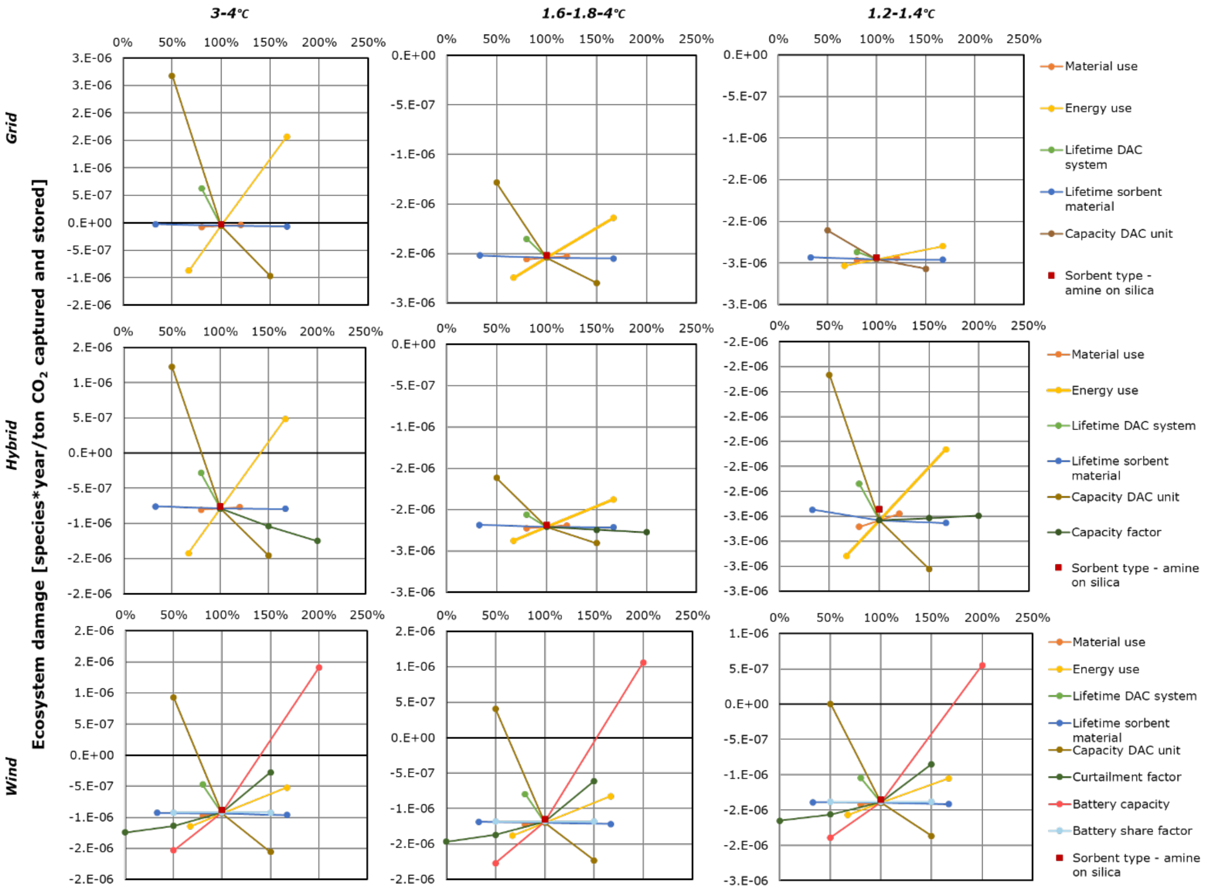


**Figure S7.** Sensitivity analysis for ecosystem damage in species*year per ton $CO_2$ captured and stored on the most uncertain parameters in the prospective assessment for all three electricity generation configurations and all three background scenarios. The results are calculated with ReCiPe 2016 (H).

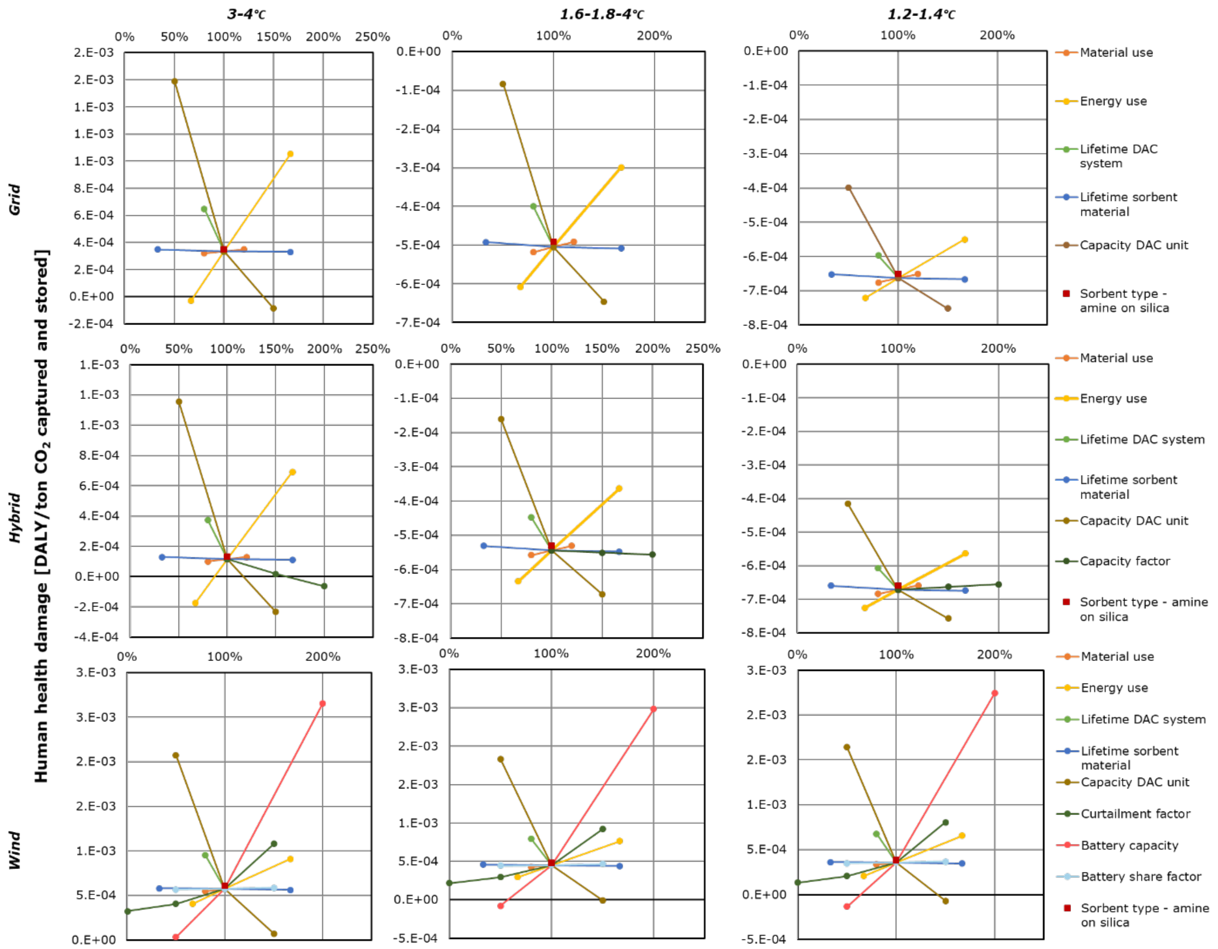


**Figure S8.** Sensitivity analysis for human health damage in DALY per ton $CO_2$ captured and stored on the most uncertain parameters in the prospective assessment for all three electricity generation configurations and all three background scenarios. The results are calculated with ReCiPe 2016 (H).

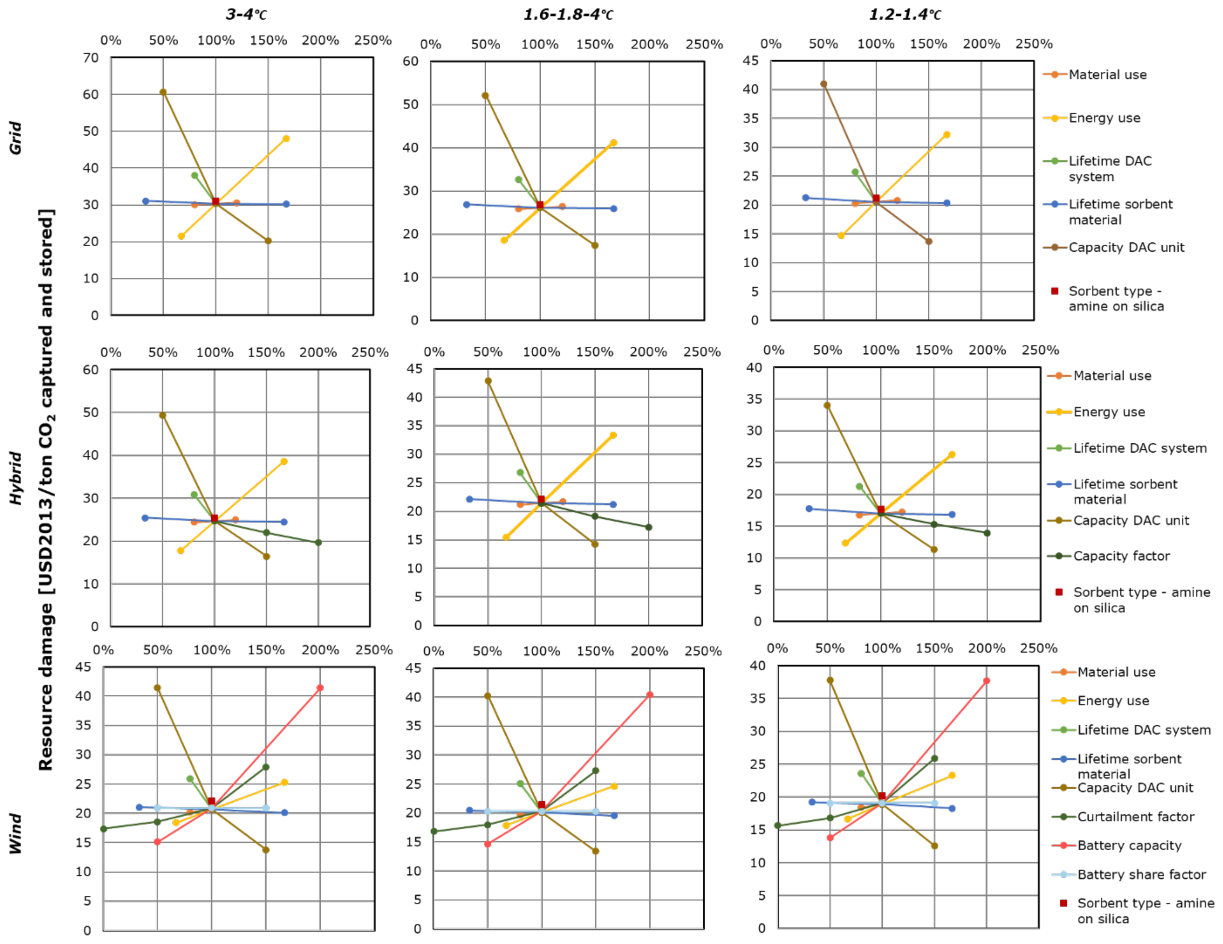


**Figure S9.** Sensitivity analysis for resource damage in USD2013 per ton $CO_2$ captured and stored on the most uncertain parameters in the prospective assessment for all three electricity generation configurations and all three background scenarios. The results are calculated with ReCiPe 2016 (H).

# References


1. Sacchi, R. *et al.* PRospective EnvironMental Impact asSEment (premise): A streamlined approach to producing databases for prospective life cycle assessment using integrated assessment models. *Renew. Sustain. Energy Rev.* **160,** 112311 (2022).

2. Huijbregts, M. *et al.* ReCiPe 2016 V1.1: A harmonized life cycle impact assessment method at midpoint and endpoint level Report I: Characterization. *ReCiPe 2016 Een geharmoniseerde levenscyclus impact Assess. methode op 'midpoint' en 'endpoint' Niv. Rapp. 1 karakterisatie* at https://rivm.openrepository.com/bitstream/10029/620793/3/2016-0104.pdf (2017)

3. Muñoz, I. & Schmidt, J. H. Methane oxidation, biogenic carbon, and the IPCC's emission metrics. Proposal for a consistent greenhouse-gas accounting. *Int. J. Life Cycle Assess.* **21,** 1069–1075 (2016).

4. Sand, M. *et al.* A multi-model assessment of the Global Warming Potential of hydrogen. *Commun. Earth Environ.* **4,** 203 (2023).
