# Supplementary material for: Prospective environmental burdens and benefits of fast-swing direct air carbon capture and storage
Source: Sci Rep. 2024 Jul 17;14:16549. doi: 10.1038/s41598-024-66990-2 (PMC11255244; doi:10.1038/s41598-024-66990-2)
Supplement: Supplementary file 3 — Supplementary Legends. [file 41598_2024_66990_MOESM3_ESM.docx]

Supplementary information 1: Supplementary methods, extra figures for damage to human health and damage to ecosystems and results for other environmental indicators (PDF).

Supplementary information 2: life cycle inventory, calculations, adjusted characterization factors and results (excel).
